# Young Tomato Plants Respond Differently under Single or Combined Mild Nitrogen and Water Deficit: An Insight into Morphophysiological Responses and Primary Metabolism

**DOI:** 10.3390/plants12051181

**Published:** 2023-03-05

**Authors:** Joana Machado, Marta W. Vasconcelos, Cristiano Soares, Fernanda Fidalgo, Ep Heuvelink, Susana M. P. Carvalho

**Affiliations:** 1 GreenUPorto—Sustainable Agrifood Production Research Centre/Inov4Agro, DGAOT, Faculty of Sciences, University of Porto, Campus de Vairão, Rua da Agrária 747, 4485-646 Vairão, Portugal; joana.machado@fc.up.pt; 2 CBQF—Centro de Biotecnologia e Química Fina—Laboratório Associado, Escola Superior de Biotecnologia, Universidade Católica Portuguesa, Rua Diogo Botelho 1327, 4169-005 Porto, Portugal; 3 Horticulture and Product Physiology Group, Department of Plant Sciences, Wageningen University, P.O. Box 16, 6700 AA Wageningen, The Netherlands; 4 GreenUPorto—Sustainable Agrifood Production Research Centre/Inov4Agro, Department of Biology, Faculty of Sciences, University of Porto, Rua do Campo Alegre s/n, 4169-007 Porto, Portugal

**Keywords:** combined abiotic stresses, gene expression, N-metabolism, osmoregulation, *Solanum lycopersicum*

## Abstract

This study aimed to understand the morphophysiological responses and primary metabolism of tomato seedlings subjected to mild levels of nitrogen and/or water deficit (50% N and/or 50% W). After 16 days of exposure, plants grown under the combined deficit showed similar behavior to the one found upon exposure to single N deficit. Both N deficit treatments resulted in a significantly lower dry weight, leaf area, chlorophyll content, and N accumulation but in a higher N use efficiency when compared to control (CTR) plants. Moreover, concerning plant metabolism, at the shoot level, these two treatments also responded in a similar way, inducing higher C/N ratio, nitrate reductase (NR) and glutamine synthetase (GS) activity, expression of RuBisCO encoding genes as well as a downregulation of *GS2.1* and *GS2.2* transcripts. Interestingly, plant metabolic responses at the root level did not follow the same pattern, with plants under combined deficit behaving similarly to W deficit plants, resulting in enhanced nitrate and proline concentrations, NR activity, and an upregulation of *GS1* and *NR* genes than in CTR plants. Overall, our data suggest that the N remobilization and osmoregulation strategies play a relevant role in plant acclimation to these abiotic stresses and highlight the complexity of plant responses under a combined N+W deficit.

## 1. Introduction

Water scarcity and nitrogen (N) over usage are two of the most serious bottlenecks to the sustainability of the agricultural sector [1]. This sector uses about 80% of the total available freshwater (which is becoming increasingly scarce), as well as high amounts of N fertilizer, with their total required amounts projected to double by 2050 [2,3,4]. However, in general, crops only take up 40–50% or less of the applied N [5,6,7], with the rest being lost to the environment consequently leading to a negative impact on soil, groundwater and freshwater bodies, ecosystems, and climate [6,8]. To enhance the sustainability of food production systems, the European Green Deal (2020) has established as targets for 2030 a reduction by at least 20% in the use of fertilizers and by 50% in nutrient losses in an attempt to contribute to reducing N leaching and improving water quality [9].

Tomato (*Solanum lycopersicum*) is one of the most cultivated vegetable crops worldwide [10]. In Europe, tomato production was valued at Euro 7.3 billion in 2017, accounting for 21% of the value of the total fresh vegetable production [11]. However, its production relies on high fertilizer inputs and regular irrigation for maximum yield [12], with most commercial tomato cultivars being quite sensitive to water deficit and N deficiency at all stages of plant development [10,13]. Thus, considering the increased consumption of fresh tomato and tomato-derived products [11], the development of strategies focused on the rational use of water and fertilizers in tomato production is highly required.

Several studies have demonstrated that both stresses, when individually imposed, negatively impact leaf and root morphological traits, affecting overall plant growth rates (recently reviewed by Machado et al. [14]). Furthermore, both stresses are known to have a severe impact on metabolic pathways, including osmotic adjustment, decreased nutrient uptake, and photosynthesis, either due to stomatal closure and/or metabolic damage [15,16]. Among the osmolytes produced, proline is known to accumulate at high levels in response to osmotic and many other abiotic stresses, including N deficiency [17,18,19]. Beyond that, several enzymes and genes involved in N metabolism and assimilation are known to be affected by N or water deficit [20,21,22,23]. N assimilation in plant tissues requires the reduction of nitrate (NO_3_^−^) by nitrate reductase (NR; EC 1.7.1.1), generating nitrite (NO_2_^−^), which is further enzymatically reduced to ammonium (NH_4_^+^). This last form can be directly assimilated by glutamine synthetase (GS; EC 6.3.1.2) into glutamine or be oxidized back to NO_2_^−^ [24]. The bioavailability of N in soil depends on the water status [25]; therefore, under drought, N uptake can be reduced in some crops (reviewed by Gonzalez-Dugo et al. [15]), including tomato [26]. In line with this, Sánchez-Rodríguez et al. [27] reported that moderate water deficit also decreased the NO_3_^−^ content in tomato cultivars sensitive to drought, as well as NR activity. This decrease in tomato NR activity was also reported by Hayat et al. [18], suggesting that both factors can interact with each other and that their combined action may induce a different plant response [15,16,23].

Indeed, recent studies have shown that plants’ physiological and molecular responses to a number of combined stresses (namely chilling and drought, drought and heat, or salinity and heat) can be unique and contrasting to those observed under single stresses [28,29,30]. Machado et al. [31] also found that tomato plants were able to employ different acclimation strategies to cope with combined N and water deficits, resulting in a significantly higher total antioxidant capacity and enzymatic activity compared to single deficits. Nonetheless, although the effect of single N and water deficits have been broadly studied in different species, research focused on the effect of combined N and water deficit is still very scarce but urgently needed to promote the efficient use of these two highly valuable crop inputs [31,32].

Based on these premises, the present study aims at contributing to a better understanding of the mechanisms behind the acclimation strategies of young tomato plants when subjected to single or combined N and water deficit. A focus was given to the morpho-physiological responses and the primary plant metabolism through the analysis of a large number of parameters, including (i) morphological traits; (ii) physiological responses (water relations, stomatal responsiveness; (iii) plant metabolism [concentration of proline, total soluble protein, N, C, and NO_3_^−^ as well as N metabolism-related enzymes (NR and GS activity)]; and (iv) transcript accumulation of C (*rcbL* and *rcbS*) and N (*GS1, GS2.1, GS2.2,* and *NR*) metabolism-related genes. It is our hypothesis that there is a cross-talking between N and water uptake; therefore, plants will respond differently to single deficits compared to combined deficits.

## 2. Results

### 2.1. Plant Growth and Physiological Parameters

Exposing young tomato plants for 16 days to single or combined mild N and water (W) deficit significantly impacted several morphological traits (Table 1). For instance, total dry weight (DW) and shoot DW were significantly lower in plants subjected to single or combined N deficit (resulting in a 27% decrease in total DW and up to 32% decrease in shoot DW compared to CTR plants). Plants exposed to single W deficit did not differ significantly from the CTR plants in those traits. In terms of root DW, no significant effects were found when comparing the CTR plants with the ones grown under different deficits. Taking into account the dry matter partitioning, plants under W deficit (single or combined) significantly invested more in their roots (leading to 30% and 33% lower shoot-to-root ratio compared to CTR plants) and reduced the partitioning towards their leaves (resulting in significantly lower leaf weight ratio). Moreover, plants grown under N deficiency (single or combined) showed a significantly lower leaf area (31 and 36% decrease) and leaf chlorophyll content (14% and 18% decrease) when compared to CTR plants (Table 1). In contrast, leaf area ratio and specific leaf area did not significantly change with any of the imposed deficits (Table 1). Additionally, plant height and its components (internode number and average internode length), as well as root length, did not respond to any of the studied deficits.

Finally, concerning the indicators of N and W use capacity, significant differences were found in the former. Plants subjected to N and N+W deficit had up to 50% lower N accumulation and up to 47% higher N use efficiency (NUE) than plants grown under CTR or W deficit conditions. The N accumulation of plants subjected to W deficit was 39% and 120% higher than in CTR and N+W deficit plants (Table 1). Water use efficiency (WUE) and shoot water potential were not significantly influenced by the studied N and W deficits (Table 1).

**Figure 1:**
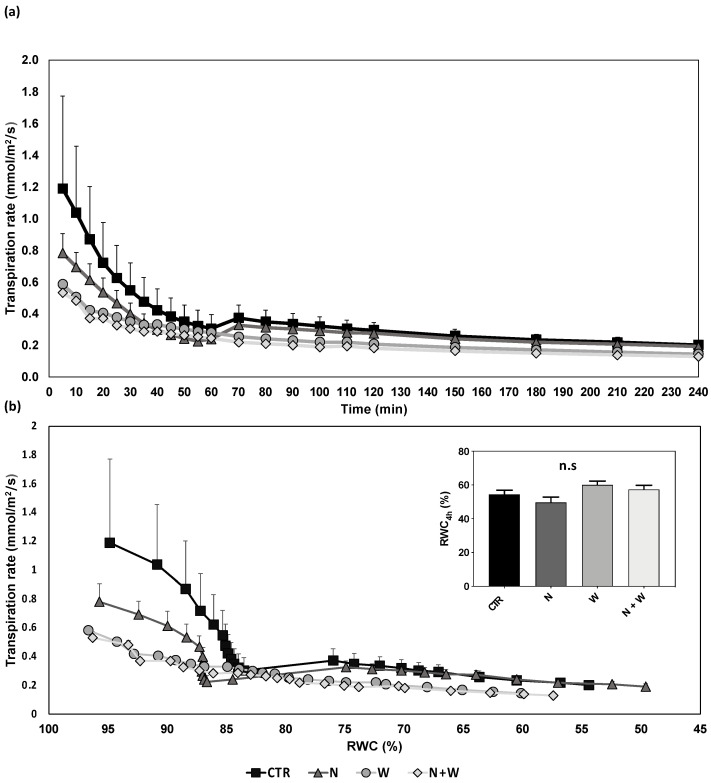
Transpiration rate as a function of time (**a**) and of relative water content (**b**) (RWC; **insert**) in detached leaflets of tomato cv. Micro-Tom grown for 16 days under control (CTR; black; 10.5 mM N + 100% W), nitrogen deficit (N; dark grey; 5.3 mM N + 100% W), water deficit (W; light grey; 10.5 mM N + 50% W) or combined N and water deficit (N+W; white; 5.3 mM N + 50% W). Data presented are mean ± SEM (n = 6). n.s. above bars indicate nonsignificant differences according to Tukey’s HSD test (*p* = 0.05).

When analyzing the transpiration rate dynamics during 4 h of leaflet desiccation, it was found that plants grown under water shortage conditions (single or combined) had a lower initial transpiration rate (up to 55%), particularly in comparison with plants grown under CTR conditions (Figure 1a). Nonetheless, the CTR plants showed a higher rate of stomatal closure in response to leaf desiccation (Figure 1b), which explains the lack of significant differences among treatments concerning the RWC after 4 h of leaf desiccation (Figure 1b, insert).

### 2.2. Plant Metabolism

#### 2.2.1. Proline and Protein Concentration

**Figure 2:**
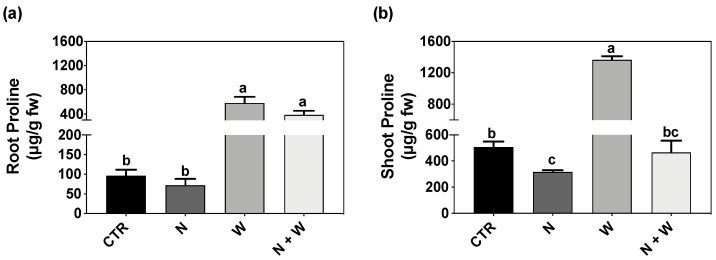
Proline concentration in roots (**a**) and shoots (**b**) of tomato cv. Micro-Tom grown for 16 days under control (CTR; 10.5 mM N + 100% W), nitrogen deficit (N; 5.3 mM N + 100% W), water deficit (W; 10.5 mM N + 50% W) or combined nitrogen and water deficit (N+W; 5.3 mM N + 50% W). Data presented are mean ± SEM (n = 3). Different letters above bars indicate significant differences according to Tukey’s HSD test (*p* = 0.05).

Proline concentration in the roots was considerably higher in plants grown under single (504%) or combined (298%) W deficit in relation to CTR, but with no significant differences between them (Figure 2a). In the shoots, the concentration of this osmolyte was only significantly increased in plants subjected to a W deficit, resulting in up to 193% higher levels compared to CTR or N+W deficit plants. In contrast, young tomato plants grown under N deficit showed 41% lower proline concentration compared to CTR conditions (Figure 2b).

**Figure 3:**
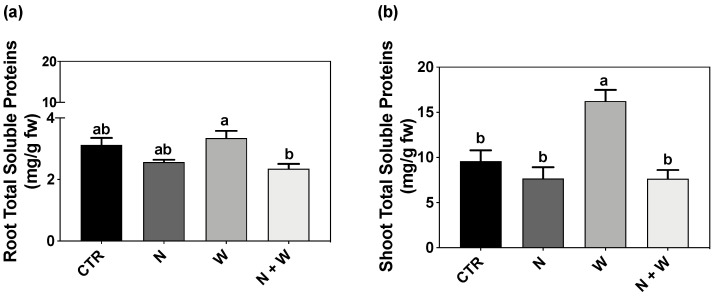
Total soluble protein concentration of roots (**a**) and shoots (**b**) of tomato cv. Micro-Tom grown for 16 days under control (CTR; 10.5 mM N + 100% W), nitrogen deficit (N; 5.3 mM N + 100% W), water deficit (W; 10.5 mM N + 50% W) or combined nitrogen and water deficit (N+W; 5.3 mM N + 50% W). Data presented are mean ± SEM (n = 3). Different letters above bars indicate significant differences according to Tukey’s HSD test (*p* = 0.05).

Total soluble protein concentration was significantly lower in the roots of plants exposed to N+W deficit compared to W deficit (30% decrease) (Figure 3a). Shoot protein concentration was significantly higher for plants grown under W deficit (70% and 113% higher compared to CTR and N+W-deficit plants, respectively), whereas the other treatments did not differ from each other (Figure 3b).

#### 2.2.2. Total Nitrogen (N), Carbon (C), Nitrate (NO_3_^−^) Quantification

**Figure 4:**
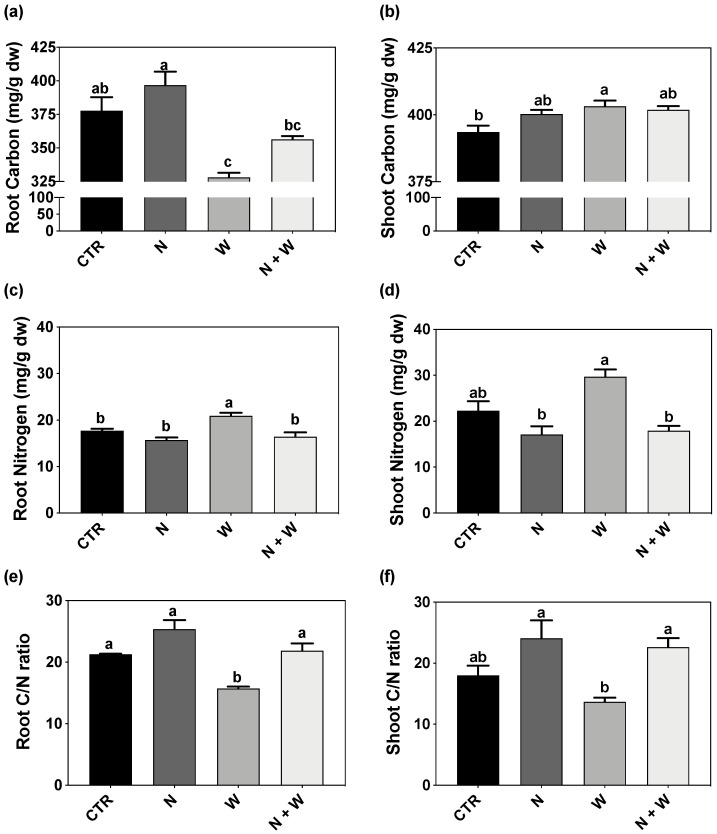
Total carbon concentration in roots (**a**) and shoots (**b**), total nitrogen concentration in roots (**c**) and shoots (**d**), and carbon to nitrogen (C/N) ratio of roots (**e**) and shoots (**f**) of tomato cv. Micro-Tom grown for 16 days under control (CTR; 10.5 mM N + 100% W), nitrogen deficit (N; 5.3 mM N + 100% W), water deficit (W; 10.5 mM N + 50% W) or combined nitrogen and water deficit (N+W; 5.3 mM N + 50% W). Data presented are mean ± SEM (n = 3). Different letters above bars indicate significant differences between treatments according to Tukey’s HSD test (*p* = 0.05).

Root C concentration reached the lowest level in W-deficit plants, having 13% less C than CTR plants (Figure 4a). Contrarily, in the shoots, the highest C concentration was found in W deficit (Figure 4b). Single W deficit led to the highest levels of N concentration both in roots and shoots (18% and 33% higher, respectively, compared to CTR) (Figure 4c,d). Furthermore, when analyzing C and N balance, it was shown that the C/N ratio reached the lowest values in the roots and shoots of W-deficit plants, decreasing by 28% and 39%, respectively, when compared to the combined N+W deficit (Figure 4e,f).

**Figure 5:**
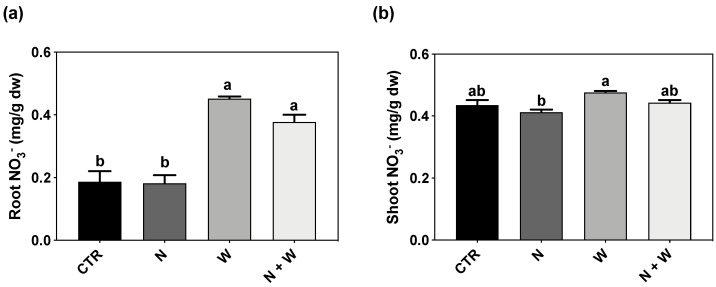
Nitrate (NO_3_^−^) concentration in roots (**a**) and shoots (**b**) of tomato cv. Micro-Tom grown for 16 days under control (CTR; 10.5 mM N + 100% W), nitrogen deficit (N; 5.3 mM N + 100% W), water deficit (W; 10.5 mM N + 50% W) or combined nitrogen and water deficit (N+W; 5.3 mM N + 50% W). Data presented are mean ± SEM (n = 3). Different letters above bars indicate significant differences according to Tukey’s HSD test (*p* = 0.05).

W deficit alone or combined with N deficit significantly increased root NO_3_^−^ concentration (137 and 100% increase in relation to CTR, respectively). The effects in the NO_3_^−^ concentration at the shoot level were far less pronounced, with no significant differences found between N+W treatment and the respective single deficits (Figure 5b).

#### 2.2.3. Nitrate Reductase and Glutamine Synthetase Activity

**Figure 6:**
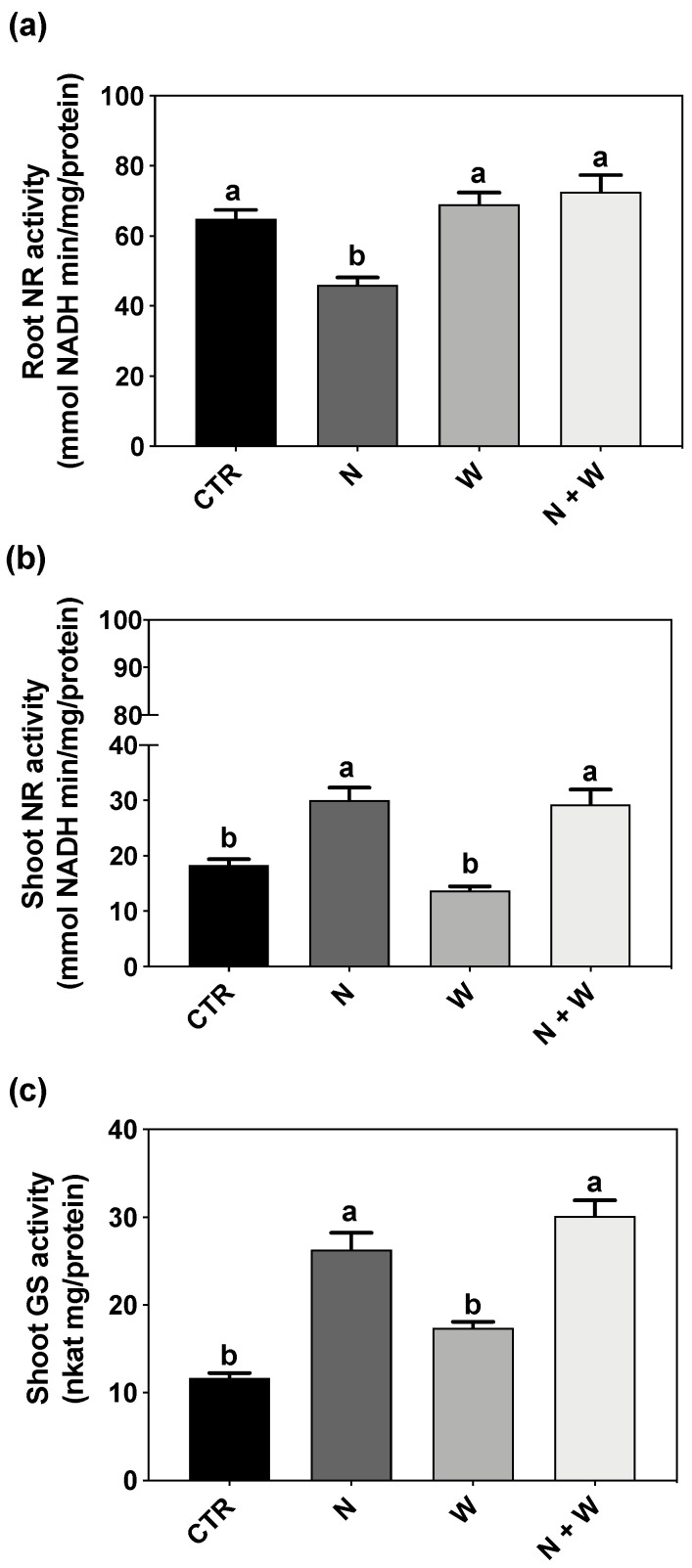
Nitrate reductase (NR) activity in roots (**a**) and shoots (**b**) and glutamine synthase (GS) activity in shoots (**c**) of tomato cv. Micro-Tom grown for 16 days under control (CTR; 10.5 mM N + 100% W), nitrogen deficit (N; 5.3 mM N + 100% W), water deficit (W; 10.5 mM N + 50% W) or combined nitrogen and water deficit (N+W; 5.3 mM N + 50% W). Data presented are mean ± SEM (n = 3). Different letters above bars indicate significant differences according to Tukey’s HSD test (*p* = 0.05). GS activity was not detectable in roots (i.e., below the limit of detection of the method).

N metabolism and assimilation were evaluated by the quantification of the total activity of NR and GS. Under N deficit, root NR activity was significantly lower as compared to all the other growth conditions (e.g., 29 and 37% decreases in relation to CTR and N+W, respectively; Figure 6a). However, in the shoots, the activity of this enzyme did not differ in plants grown under single or combined N deficit, although both treatments presented significantly higher enzyme activity (up to 119%) when compared to CTR and W-deficit plants (Figure 6b).

The GS activity in the roots was below the limit of detection for all treatments, whereas in the shoots, it was found the same pattern as the shoot NR activity, i.e., with the W-deficit plants being similar to CTR plants and N+W plants having a similar shoot GS activity compared to the N-deficit plants (Figure 6c).

#### 2.2.4. RuBisCO and N Metabolism-Related Transcript Accumulation

**Figure 7:**
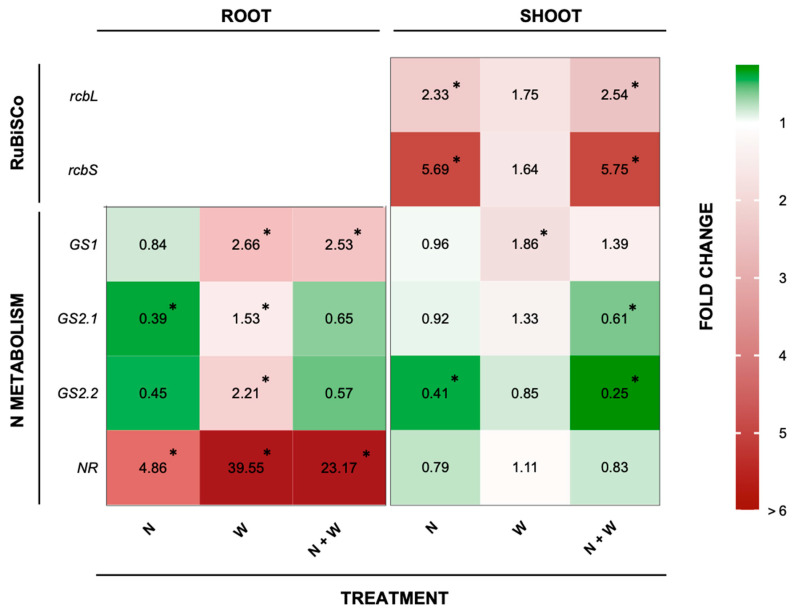
Relative fold of expression of RuBisCO and N metabolism-related genes in tomato cv. Micro-Tom roots and shoots as affected by nitrogen deficit (N; 5.3 mM N + 100% W), water deficit (W; 10.5 mM N + 50% W), or combined nitrogen and water deficit (N+W; 5.3 mM N + 50% W). The 2^−∆∆Ct^ values against the control are shown. * above mean values indicate significant differences compared to the control according to the t-test (*p* = 0.05). Green indicates downregulation and red upregulation compared to the control plants. White indicates that gene expression was maintained at levels identical to the CTR.

Among the two studied RuBisCO metabolism-related genes, *rcbS* was the most affected. Its expression level was upregulated by 5.7 and 5.8-fold in plants subjected to N or N+W deficit, respectively, compared to the CTR (Figure 7). *rcbL* expression followed the same pattern but with a lower magnitude of change, resulting in an upregulation of 2.3-fold and 2.5-fold when plants were exposed to single or combined N deficit (Figure 7). In contrast, under W deficit none of these gene expression patterns were significantly impacted (Figure 7).

*GS1* expression in the roots was significantly higher in plants grown under W and N+W deficit (2.7- and 2.5-fold, respectively). In shoots, *GS1* transcriptional levels were only significantly higher in plants exposed to W deficit (1.9-fold), but *GS2.1* was significantly downregulated in roots of plants grown under N shortage (0.4-fold for N and 0.7-fold for N+W plants). Moreover, W deficit upregulated *GS2.2* in roots (2.2-fold increase) (Figure 7), while in shoots it was downregulated in plants suffering from N and N+W deficit (0.4 and 0.3-fold). From the studied genes, *NR* was the most affected in roots (Figure 7). W deficit led to the highest upregulation (an increase of 40-fold), followed by N+W (23-fold increase) and N deficit (an increase of 4.9-fold).

### 2.3. Principal Component Analysis

**Figure 8:**
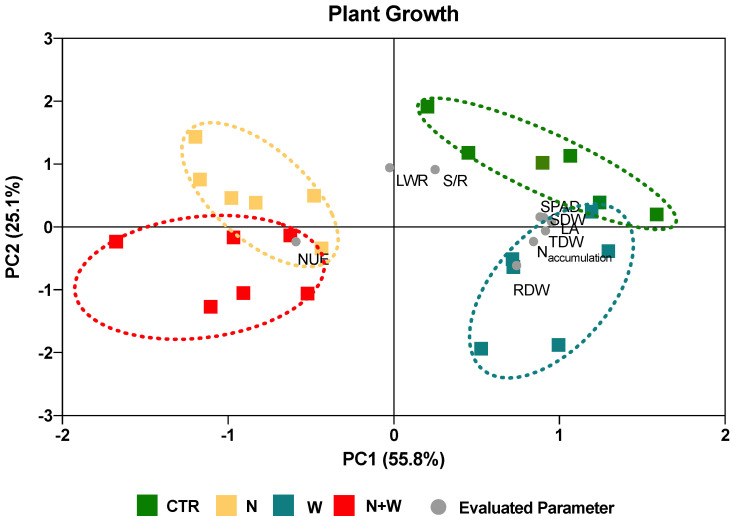
Principal component analysis (PCA) of the plant growth parameters evaluated in tomato cv. Micro-Tom grown for 16 days under control (CTR; 10.5 mM N + 100% W), nitrogen deficit (N; 5.3 mM N + 100% W), water deficit (W; 10.5 mM N + 50% W) or combined nitrogen and water deficit (N+W; 5.3 mM N + 50% W). The two principal components (PC1, PC2) explained 80% of the total variance found among treatments. Details on the abbreviations are given in Material and Methods.

**Figure 9:**
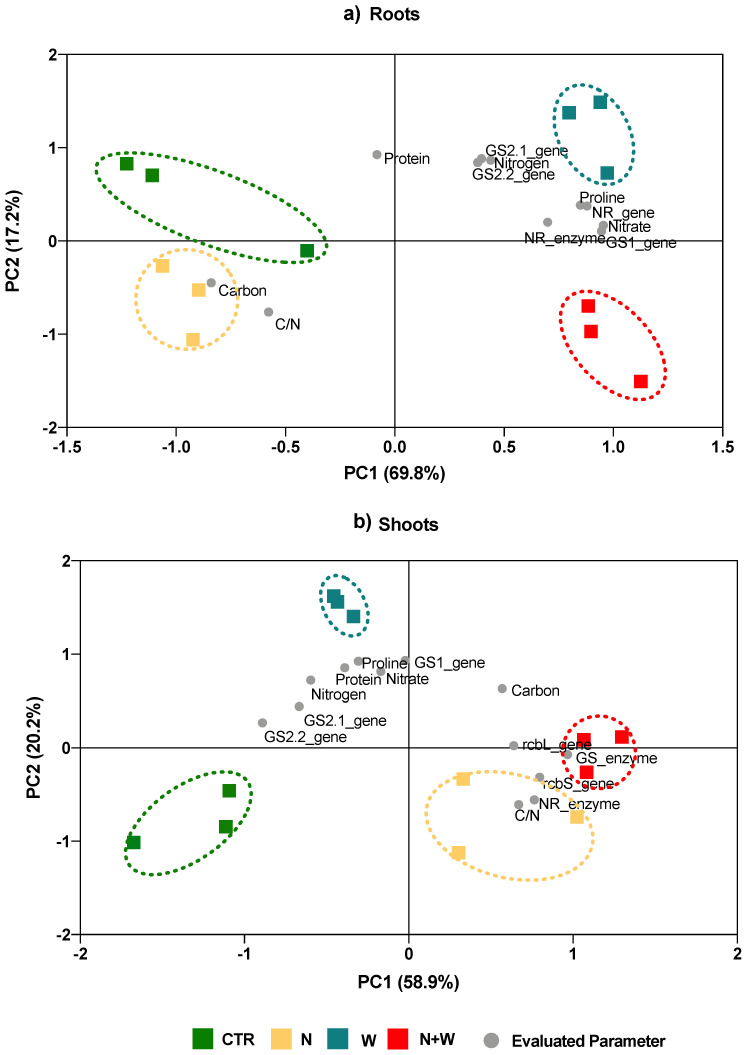
Principal component analysis (PCA) of the plant metabolism in roots (**a**) and shoots (**b**) evaluated in tomato cv. Micro-Tom grown for 16 days under (CTR; 10.5 mM N + 100% W), nitrogen deficit (N; 5.3 mM N + 100% W), water deficit (W; 10.5 mM N + 50% W) or combined nitrogen and water deficit (N+W; 5.3 mM N + 50% W). The two principal components (PC1, PC2) explained 87% and 79% of the total variance found among treatments in roots and shoots, respectively. Details on the abbreviations are given in Material and Methods.

In order to determine how all the analyzed variables explain the differences among the four treatments, a PCA was performed (Figure 8 and Figure 9). Results showed that, concerning plant growth, the two first components accounted for 56% and 25% of the variance. Moreover, N and N+W treatments shared more similarities among them than with plants from CTR and W treatments, with the former treatments being characterized by lower chlorophyll content (SPAD), shoot DW, leaf area, total DW, N assimilation, and root DW and by a higher NUE. Additionally, CTR and N deficit plants were mostly associated with higher leaf weight ratios and shoot-to-root ratios (Figure 8).

Concerning plant metabolism, the first component accounted for 70 and 59% of the variance in roots and shoots, respectively, and the second for 17 and 20%, respectively (Figure 9a,b). In the roots, CTR and N deficit were the treatments sharing more similarities, having an opposite behavior to the W and N+W deficit groups. Indeed, roots from plants grown under single or combined W deficit were associated with enhanced NO_3_^−^ and proline concentrations, NR activity, and *GS1* an *NR* expression, but with a lower C concentration (Figure 9a). The CTR and W deficit were associated with a higher *GS2.1* and *GS2.2* transcript-levels and N concentration (Figure 9a). In shoots, N and N+W groups showed a higher C/N ratio, NR and GS activity, and a higher rcbS and *rcbL* transcript-levels together with a lower N concentration and expression of *GS2.1* and *GS2.2* genes. The W deficit was closely associated with higher NO_3_^−^, proline, and protein concentrations alongside with increased transcript-levels of *GS1* (Figure 9b).

## 3. Discussion

### 3.1. Effect of Combined N and Water Deficit on Plant Growth and Physiological Parameters

It is well known that plant responses to abiotic stresses significantly differ at various organizational levels, depending upon the intensity and the duration of the imposed stress, as well as on the plant species and its developmental stage [33,34]. Although in our study we have applied a gradual and mild deficit throughout the vegetative cycle of tomato, several important growth-related traits, such as total DW, shoot DW, root DW, shoot-to-root ratio, leaf area, leaf weight ratio, chlorophyll content, NUE, and N accumulation were severely affected by at least one of the imposed deficits (Table 1, Figure 8). In contrast, some of the studied plant growth-related traits, such as plant height, root length, internode number, internode length, leaf area ratio, specific leaf area, WUE, and shoot water potential, known to be severely affected under N or drought stress [35,36,37], were not significantly affected in this study (Table 1). Nonetheless, it is expected that if the trial had been prolonged, these deficits would have become more severe and treatment effects more evident due to the longer duration of the imposed stresses.

Integrating all the studied growth traits in a PCA revealed considerable variability among treatments, with the two principal components explaining 80% of the variability (Figure 8). The PCA showed that plants exposed to N+W deficit behaved similarly to plants exposed to a single N deficit, having a lower total DW, shoot DW, root DW, leaf area, chlorophyll content, and N accumulation as well as a higher NUE. This suggests that in plants grown under a combined N+W deficit, the N deficit had a more predominant impact on the studied growth traits than the W deficit (Figure 8). Additionally, W-deficit plants were more similar to plants grown under CTR conditions (Figure 8), suggesting that the level of water deficit imposed in this study was not severe enough to negatively impact the growth of young tomato plants.

Due to the stronger negative effect of water deficit on shoots than root growth, the shoot-to-root ratio tends to decrease with drought [13,38]. Because roots contain much lower concentrations of N than aboveground biomass, Gonzalez-Dugo et al. [15] suggest that this change in the allocation pathway diminishes N needs. Our results agree with such hypothesis, as the highest decreases in the shoot-to-root ratio were observed in plants subjected to combined N+W deficit. Additionally, several parameters related to leaf growth, namely leaf number, leaf area, leaf area ratio, and leaf weight ratio, were also reported to be significantly lower in tomato plants exposed to W deficit or N deficit [14,39,40,41]. In our study, a decrease in leaf weight ratio was observed upon exposure to W and N+W deficit plants, while leaf area was reduced under N and N+W deficit (Table 1). When facing N limitation, plants can reduce their leaf area, paired with small changes in leaf N concentration, or alternatively, can maintain their leaf area and adapt leaf N concentration to N availability [42]. Based on the collected data, our results pointed out to the first strategy, as a decrease in leaf area was observed, but no significant differences in leaf N concentration were found in plants subjected to N and N+W deficit compared to CTR plants (Table 1 and Figure 4b). Additionally, chlorophyll content, which is considered a key indicator of the photosynthetic capacity [43], is also often substantially decreased after drought exposure [44] or when plants face N deficiency due to N partitioning dependence of the photosynthetic machinery [45]. In the present work, total chlorophyll content was not significantly affected by W deficit but was significantly decreased by N deficit and N+W deficit (Table 1). Other studies also reported that, despite the stress condition, tomato plants did not significantly change their chlorophyll content, as observed in other drought-exposed species [34,46,47]. Moreover, no evident signs of leaf chlorosis were macroscopically detected, but the negative effects of N deficit on total chlorophyll content were evident (Table 1). Soval-Villa et al. [48] reported that N status may be indirectly determined by the chlorophyll concentration present in leaves, even at early stages, as it is directly correlated to their N concentration. Thus, we can assume that in this study plants exposed to N deficit (single or combined) were starting to develop clear N deficiency symptoms.

Furthermore, NUE, an important parameter to benchmark N management, which is highly dependent on N-remobilization efficiency from senescing leaves [49], and a crucial factor behind early leaf senescence [50], was significantly increased by N and N+W deficit (Table 1). This higher NUE could have resulted from the ability of the young tomato plants to efficiently remobilize N as suggested by the lower chlorophyll content (Table 1), lower N accumulation (Table 1), higher C/N ratio (Figure 4e,f; Figure 9a,b), and the higher activity of N metabolism-related enzymes in shoots (Figure 6b,d and Figure 9b). Sánchez-Rodríguez et al. [51] reported that water deficit provokes a decline in the concentration and accumulation of N in four commercial cherry tomato cultivars grown under W deficit but not in the tolerant cv. Zarina, where an improvement was found. Our results are in accordance with those observations as we also found significantly higher N accumulation in plants exposed to W deficit (Table 1, Figure 4a,b). Furthermore, mild W deficit was reported to promote root growth and consequently nutrient uptake [52], which can be seen in this study as a tendency for longer roots and significantly higher root dry weight (Table 1). Actually, the stimulation of root length is a common response of plants to water deprivation in an attempt to reach deeper soil layers to search for additional water and nutrients [53]

### 3.2. Effect of Combined N and W Deficit on Plant Metabolism

The accumulation of solutes to decrease water potential (i.e., osmotic adjustment) is one of the most common responses of plants to maintain the water potential gradient as the soil becomes drier [54,55,56]. Of these solutes, the most conspicuous accumulation is of proline. Furthermore, proline not only acts as an osmolyte but also as an antioxidant and a nutritional source, being used as a metabolic-compatible solute to store and transfer N and C after oxidation [17,18]. In our study, proline levels were also significantly increased both in roots and shoots of plants grown under W deficit but decreased in shoots of plants subjected to single N deficit. When the N deficit was simultaneously applied with the water deficit, proline levels decreased in relation to the single W deficit yet presented higher levels than in plants only exposed to the N deficit (Figure 2). This result is in accordance with Szabados and Savouré [17], who showed that proline accumulates as a compatible osmolyte during water deficit and then is used as a readily accessible source of N in order to compensate for the N deficit. Interestingly, while in roots of N+W deficit plants, the behavior was more similar to W deficit plants, in shoots, N+W deficit plants were more comparable to those grown under the single N deficit (Figure 2 and Figure 9a,b).

Variation in protein content is another essential part of plants’ acclimation to changes in environmental conditions [57,58]. Often, to tolerate drought stress, plants accumulate soluble proteins which help to stabilize the structure of cells and proteins [59]. In addition, drought stress often leads to altered water homeostasis within the plant, which can be balanced through the enhancement of water passage through protein membranes, in which aquaporins have a major role [60,61]. In our study, W deficit significantly increased shoot soluble protein concentration (Figure 3b). This suggests an accumulation of drought-induced proteins, which might be somehow related to the above-mentioned physiological processes as an acclimation response to water limitation, which could improve plant drought tolerance, thus contributing to the absence of severe effects on the morphological and physiological evaluated traits [62]. On the other hand, N deficiency accelerates leaf senescence and the production of reactive oxygen species, leading to the degradation of macromolecules like proteins and chlorophylls. Due to the pivotal role of N in protein biosynthesis, N deficiency can reduce amino acids and protein content, which has been observed in tomato [63,64]. In our study, both roots and shoots of N and N+W deficit plants tended to have a lower concentration of total soluble proteins (Figure 3). However, only in plant roots exposed to the combined N+W deficit the total soluble protein content was significantly lower (Figure 3a), once again suggesting that stress combinations can induce unique responses that cannot be simply extrapolated from the exposure to the single deficits.

Although under drought conditions, N uptake usually diminishes [15], in our study, surprisingly, the N accumulation and concentration increased both in roots and shoots of plants exposed to W deficit (Table 1, Figure 4c,d). This is in accordance with the higher amino acids (proline) and total soluble proteins that we also observed in these plants (Figure 2 and Figure 3). Indeed, drought may increase soil solute concentration, which promotes nutrient uptake [15,16,23]. Furthermore, the availability of C and N is an important factor for the regulation of plant metabolism and development; however, the ratio of C to N metabolites in the cell has a higher role in the regulation of plant growth and development than C or N individually [65,66]. Leaf senescence can be triggered by high C and low N availability (a typical C/N imbalance) [67]. In our study, the C/N ratio significantly decreased under the W deficit, disrupting the C/N balance, especially in the roots (Figure 4a,b). However, the C/N ratio tended to increase in plants exposed to N or N+W stress (Figure 4e,f). This tendency to increase the C/N ratio suggests that leaf senescence may have occurred, as previously reported by Aoyama et al. [67].

Furthermore, under drought conditions, transpiration and turgor losses may cause a decrease in NO_3_^−^ absorption by the roots, as well as a reduction in the translocation from the roots to the leaves [68,69]. In our study, NO_3_^−^ concentration increased in roots exposed to W deficit but not in shoots (Figure 5). Sánchez-Rodríguez et al. [27] reported that under moderate water stress conditions, NO_3_^−^ significantly diminished in some tomato cultivars but increased in others, suggesting that this trait might follow a cultivar-dependent response. Moreover, drought stress also restricts the ability of plants to reduce and assimilate N through the inhibition of enzymes involved in N metabolism and assimilation, such as NR and GS [70,71]. However, as with NO_3_^−^ concentration, we did not detect significant changes in NR activity in plants grown under W deficit (Figure 6b). Nevertheless, this group of plants presented a significantly higher NR transcript-level than the CTR plants (Figure 7). As the expression of NO_3_^−^ assimilation genes is highly controlled by NO_3_^−^ concentration [20], the significant upregulation found in roots of W and N+W-deficit plants is probably correlated with the significant increase in NO_3_^−^ concentration observed in roots of plants subjected to water shortage (Figure 5a and Figure 7). However, as proteolytic activity increases during exposure to water stress, enhanced protease activity has been implicated in the acceleration of protein turnover, with typical proteinogenic amino acids and proline accumulation occurring in water-stressed plants [72]. Accordingly, in our study, the upregulation in the *NR* gene was not translated into a clear increase in the enzyme activity (Figure 6a and Figure 7), but increased levels of proline were observed (Figure 3). Thus, we hypothesize that the increased NO_3_^−^ concentration served as an intracellular signal to induce a higher expression of NR coding genes, but drought-induced effects, probably on enzyme activity and stability, did not allow a higher catalytic activity of this enzyme. Concerning plants grown under N shortage, NO_3_^−^ concentration was not affected in plants grown under the single N deficit, but it was increased in roots of plants subjected to the combined N+W deficit, to the same extent as that of plants grown under single W deficit (Figure 5a). The activity of the NR enzyme is increased in the presence of low NO_3_^−^ concentrations and starts to decrease at higher cytoplasmic NO_3_^−^ levels [20]. Therefore, it is not surprising that low levels of external NO_3_^−^ have a positive effect on NR activity [20]. In line with this significantly lower NO_3_^−^ concentration (Figure 5a), the upregulation of *NR* observed in roots of plants grown under N deficit was considerably lower than in plants grown under W and N+W combined deficit (Figure 7). In contrast, in plants grown under N deficit, loss of NR protein occurred even when an increase in *NR* transcript-level was found (Figure 6a and Figure 7). This can be explained, for example, by the induction of NR proteases that can occur due to high levels of sugars [73], which are usually found under N deficiency conditions [74]. In tomato and tobacco plants, high NO_3_^−^ levels also induced an increase in the level of NR mRNA after N starvation, followed by a decrease in NR protein and NR activity [75], which agrees with our results (Figure 6a and Figure 7). In shoots, NR regulation was maintained in the basal threshold, which is also in line with the shoot NO_3_^−^ concentration that we found (Figure 5b and Figure 7). In this organ, NR seems to be controlled post-transcriptionally as no alteration in its expression was detected, but there was an increased activity of the enzyme (Figure 6b and Figure 7).

In line with NR, GS activity was not affected in plants subjected to W deficit (Figure 6d). The same was previously observed in some tomato cultivars subject to mild water deficit [27]. On the other hand, GS has an important role in senescence-induced nutrient remobilization (reviewed by Masclaux-Daubresse et al. [76]). In Arabidopsis and oilseed rape, it was shown that N can be remobilized from senescing leaves to expanding leaves at the vegetative stage [21,77,78]. Thus, the increase in GS activity observed in shoots of plants exposed to N and combined N+W deficits suggests a N remobilization strategy (Figure 6c). Additionally, the increased level of *GS* transcripts found in roots of W and N+W stressed plants point to an acclimation mechanism that plants used to increase GS activity, thus enhancing proline levels in response to the high levels of NO_3_^−^. Accordingly, although the GS activity in the roots was belong the limit of detection, when plants were grown under water shortage (W and N+W), increased levels of NO_3_^−^ and proline were found (Figure 2a and Figure 5a). Like for *NR*, regarding N deficit in shoots, the regulation of *GS1*, which is known to be involved in N remobilization, seems to be post-transcriptional since there was no change in gene expression, but there was a greater activity of the enzyme when plants were grown under this deficit conditions (Figure 6c and Figure 7).

RuBisCO degradation is a common response of plants exposed to abiotic stress. Together with other photosynthesis-related proteins, RuBisCO is a major source of N for remobilization. Transcriptome analysis has shown that many autophagy genes related to RuBisCO are upregulated during senescence and in response to N limitation [79,80]. In our study, *rcbS* and *rcbL* were upregulated in the presence of all stress treatments (Figure 7). However, N and N+W deficits had a higher impact, especially regarding *rcbS*, whose expression was considerably increased in both treatments. These results confirm the increase observed for C/N ratio and GS activity as well as the decrease observed in proline and total soluble protein concentration, which also points towards N remobilization. On the other hand, the evaluation of *rcbS* and *rcbL* genes also suggests that W deficit alone did not affect RubisCO activity which is aligned with the low level of stress imposed previously mentioned and pairs with the lack of differences found on the chlorophyll content (Figure 7 and Table 1).

The integration of all biochemical and gene traits linked to plant metabolism in a PCA revealed considerable variability among treatments, with the two principal components explaining 87% of the variability in roots and 79% in shoots (Figure 9a). The PCA showed that, in roots, plants under N+W deficit had a behavior similar to that of plants exposed to single W deficit, presenting higher NO_3_^−^ and proline concentrations, as well as higher NR activity and *GS1* and *NR* expression (Figure 9a). This indicates that in roots from plants grown under combined deficit, W deprivation had a higher contribution to the changes in biochemical traits and gene expression levels than N deficit (Figure 9a). Contrarily, the PCA revealed that, in shoots, plants grown under combined N+W had a behavior similar to that of plants exposed to single N deficit, having higher C/N ratio, NR and GS activity, and *rcbS* and *rcbL* transcript-levels but lower N concentration and *GS2.1* and *GS2.2* levels (Figure 9b). The differential behavior between combined N and water deficit and single deficits, as well as between plant organs (shoots and roots) was already reported in terms of enzymatic and non-enzymatic antioxidant responses [31] and highlights the need for more studies focused on plant responses to combined deficits.

## 4. Materials and Methods

### 4.1. Plant Material and Growth Conditions

The experiment was carried out in a growth chamber (temperature: 25 °C day/23 °C night; relative humidity: 70%; photosynthetic photon flux density (PPFD): 300 µmol/m^2^/s; photoperiod: 16 h light) according to a complete randomized design.

Seeds of *Solanum lycopersicum* cv. Micro-Tom were sown in trays filled with commercial germination potting substrate (SIRO, Portugal). At the third leaf stage (approximately three weeks after sowing), seedlings were selected for uniformity to be transplanted to single-plant pots (10 cm high, 10 cm diameter) filled with 0.1 to 1.5 mm-grade vermiculite (60 g). These tomato seedlings were divided into four groups for applying the following treatments: control (CTR; 10.5 mM N; 100% field capacity), nitrogen deficit (N; 5.3 mM N), water deficit (W; 50% field capacity), and combined stress (N+W; 5.3 mM N; 50% field capacity). Since we aimed at having a progressive water deficit, as seedling transplanting is a critical phase of the cultivation period, just prior to transplanting, all pots were irrigated to field capacity (FC), determined using the soil gravimetric water content method [31]. This corresponded to 300 mL of the respective nutrient solution applied to each pot [CTR and W deficit treatments: 10 mM NO_3_^−^; 0.5 mM NH_4_^+^; 1.9 mM H_2_PO_4_; 6.1 mM K^+^; 3.6 mM Ca^2+^; 1.6 mM SO_4_^2^; 2.5 mM Mg^2+^; 2.6 mM Cl^−^; 0.5 mM HCO_3_^−^ (pH of 6 and E.C. of 2.50 dS/m); N deficit and N+W deficit treatments: 5 mM NO_3_^−^; 0.3 mM NH_4_^+^; 1.9 mM H_2_PO_4_; 6.1 mM K^+^; 3.6 mM Ca^2+^; 4.8 mM SO_4_^2^; 2.8 mM Mg^2+^; and 5.5 mM Cl^−^; 0.5 mM HCO_3_^−^ (pH 6 and E.C. of 2.80 dS/m)]. Both nutrient solutions had the following micronutrient composition: 35 μM Fe-EDDHA; 10 μM Mn^2+^; 20.1 μM B; 0.9 μM Cu^2+^; and 5.0 μM Zn^2+^; 0.5 μM MoO_4_^2−^. Following seedling transplanting, all pots were covered to prevent evaporation, and no more nutrient solution was added until the end of the experimental period. Concerning further water supply, each pot from the CTR and N deficit treatments was daily weighted and rewatered (with distilled water) to maintain FC at 100% throughout the trial. In contrast, in W and N+W deficit treatments, no additional irrigation was given to the plants, resulting in a progressive decrease of substrate FC. Ten days after transplanting, five plants from each treatment were randomly collected. The average fresh weight of these plants (per treatment) was subtracted from the total pot weight (per treatment) to calculate the actual weight of the water/nutrient solution present in the pot (which corresponded to 75% FC for Treatment W deficit and N+W deficit). The experiment stopped when plants subjected to single or combined water deficits reached 50% of FC (corresponding to 16 days after transplanting).

At harvest, an analysis of the morphological and physiological parameters was performed (n = 6). The remaining nine plants were separated into roots and shoots and immediately frozen in liquid nitrogen, macerated (n = 3, each biological replicate consisting of a pool of three individual plants), and subsequently, stored at −80 °C for further use in biochemical and molecular analyses.

### 4.2. Plant Growth and Physiological Parameters

At the end of the experimental period, several plant biometric traits were measured (Table 1). The maximum root length was recorded, and total leaf number only took into account leaves with more than 1 cm in length. Total leaf area was measured using an LI-3100C area meter (LI-COR, Lincoln, NE, USA). Dry weight (DW) of leaves, stems, and roots was determined separately (48 h at 105 °C in a ventilated oven). Specific leaf area (ratio of leaf area to leaf dry weight; cm^2^/g dw), leaf weight ratio (ratio of leaf dry weight to total dry weight; g/g dw), leaf area ratio (ratio of leaf area to total dry weight; cm^2^/g dw), and shoot-to-root ratio (ratio of shoot-to-root dry weight; g/g dw) were calculated according to the “classical approach” described by Hunt [81].

Leaf chlorophyll content was measured on the youngest fully expanded leaf using the non-destructive chlorophyll meter SPAD (Konica Minolta SPAD-502 model, Minolta, Tokyo, Japan).

N use efficiency and N accumulation were calculated as follows: ratio of total plant DW to total N applied (g dw/g N applied) and coefficient between the total N concentration in all plant tissues and the total DW of the plant (mg N).

Plant water status was estimated by measuring shoot water potential (Ψ; MPa) 2 h before the photoperiod, using a Scholander pressure chamber. Water use efficiency (mg/mL) was calculated as the ratio between total plant DW (mg) and water consumption per plant (mL).

Stomatal responsiveness to leaf desiccation was assessed gravimetrically following the method described by Carvalho et al. [82]. In short, the youngest fully expanded leaflet of each plant was detached at the onset of the light period. Immediately after excision, leaflet petioles were recut under water and placed in flasks filled with degassed water. Leaflets were further incubated for 2 h to establish their saturated fresh weight. After rehydration, the leaflets were allowed to desiccate for 4 h on a bench. Leaflet weight was gravimetrically recorded every 5–10 min for 4 h. Following the evaluation of stomatal responsiveness, leaflet area (using Image J; https://imagej.nih.gov/ij/, accessed on 15 December 2021) and dry weight were measured. The transpiration rate was calculated according to Equation (1), and for the calculation of the RWC, Equation (2) was employed. In both measurements, one leaflet per plant was evaluated for six plants per treatment.

(1) $$\mathrm{Transpiration}\;\mathrm{rate}\;(\mathrm{mmol}/m^{2}/s^{1})\;=\left(\frac{\left(\Delta leaflet\;fresh\;weight\;\left(g\right)\right)}{molar\;mass\;water\;\left(g/{mol}^{}\right)}\times 1000\left(mmol/{mol}^{}\right)\right)/\;measurement\;interval\;\left(s\right)/leaflet\;area\;(m^{2})$$

(2) $$\mathrm{RWC}=\frac{fresh\;weight-dry\;weight}{saturated\;fresh\;weight-dry\;weight}\times 100$$

### 4.3. Plant Metabolism

#### 4.3.1. Proline and Protein Quantification

Proline concentration was quantified spectrophotometrically in frozen roots and shoots (250 mg), according to the method of Bates et al. [83]. Briefly, after reading sample absorbance at 520 nm, proline levels were calculated through a standard curve previously prepared with known concentrations of proline and expressed in terms of fresh weight (FW).

The extraction of total soluble protein was performed under cold conditions according to Fidalgo et al. [84], using 250 mg of frozen roots and shoots. After centrifugation, the supernatant was collected, and the soluble protein concentration of each sample was determined by the Bradford method [19]. Results were calculated from a standard curve prepared with solutions of known concentration of bovine serum albumin (BSA) and expressed on an FW basis.

#### 4.3.2. Total Nitrogen (N), Carbon (C), Nitrate (NO_3_^−^) Quantification

These analyses were performed by an external lab (A2 Análises Químicas Lda, Guimarães, Portugal). The N and C concentrations were determined using the DUMAS method based on ISO-16634-1 (ISO, 2008). The NO_3_^−^ quantification samples were macerated and extracted with water and quantified by the potentiometric method based on the methods described by Mills and Jones [85].

#### 4.3.3. Extraction and Quantification of N Metabolism-Related Enzymes

The quantification of nitrate reductase (NR; EC 1.6.6.1.) activity was performed in roots and shoots by spectrophotometry according to the method of Kaiser and Brendle-Behnisch [86]. NR activity was estimated using NADH extinction coefficient (39.4 mM/cm) and expressed as mmol/min for each mg of protein.

Glutamine synthetase (GS; EC 6.3.1.2.) activity was determined following the protocol of Shapiro and Stadtman [87], using frozen roots and shoots. The activity was determined by the transferase assay, based on a colorimetric assay, by recording the absorbance at 500 nm. After measuring the absorbance, the activity of GS was calculated and expressed as a nkat/mg of protein.

#### 4.3.4. Gene Expression Quantification Analysis of RuBisCO and N Metabolism-Related Genes

The transcript-levels of *rcbS, rcbL, GS1, GS2.1, GS2.2,* and *NR* genes were evaluated by quantitative real-time PCR in roots and shoots. Total RNA was extracted from frozen roots and shoot tissues (ca. 100 and 50 mg, respectively) using the RNeasy Plant Mini Kit (Qiagen, Hilden, Germany), according to the manufacturer’s instructions. RNA yield was quantified using a NanoPhotometer (Implen GmbH, München, Germany), and its integrity was checked by agarose gel electrophoresis. Single-stranded cDNA was synthesized using iScript cDNA Synthesis Kit (Bio-Rad, Hercules, CA, USA) according to the manufacturer’s instructions in a Doppio Thermal Cycler (VWR, Oud-Heverlle, Belgium).

Primers for *GS1, GS2.1*, and *GS2.2* genes were selected using Primer-BLAST from NCBI (https://www.ncbi.nlm.nih.gov/tools/primer-blast/, accessed on 15 January 2022) for an expected PCR product of 70–150 bp and primer melting temperatures between 57 and 63 °C, whereas primer sequences for *rcbL, rcbS,* and *NR* genes were obtained from previous studies [88,89]. The primer sequences for each gene are listed in Table 2.

Reverse transcription polymerase chain reactions (RT-qPCR) were performed on a thermal cycler CFX96 Touch Deep Well Real-Time PCR Detection System (Bio-Rad Laboratories, Inc, California, USA) and data visualized with the software Bio-Rad CFX Manager 3.1. The amplification protocol cycle was 95 °C for 3 min and 40 cycles at 95 °C for 15 s, 30 s at each primer pair optimal annealing temperature (Table 2), and 72 °C for 30 s. Amplifications were carried out using a final volume of 20 μL, which consisted of 1 μL of the primer forward and 1 μL of the primer reverse (both at 6 μM), 10 μL of 2× iQ SYBR^®^ Green Supermix (Bio-Rad, CA, USA), and 8 μL of a 1:100 dilution of the template cDNA. Melt curve profiles were analyzed for each tested gene. The comparative CT method (ΔΔCT, [90]) was used for the relative quantification of gene expression values using ubiquitin (*UBI*) and TIP41-like protein (*TIP41*) genes as control transcript and the control plants as the reference sample. For relative gene expression analysis, the fold change with biological significance was considered to be lower than 0.5 (downregulation) or higher than 2 (upregulation) [91], and for each of the three biological replicates and target genes, two technical replicates were analyzed.

### 4.4. Statistical Analysis

For each analysis performed, there was a different number of biological replicates (specified in Section 4.1). Results were expressed as mean ± standard error of the mean. Data were subjected to a one-way analysis of variance (ANOVA), followed by Tukey’s posthoc test whenever significant differences were found (*p* = 0.05). Additionally, a Principal Component Analysis (PCA) with *Varimax* rotation was performed to establish the relationship among the variables, which showed significant differences among treatments. Data included in the PCA (n = 6 for plant growth and n = 3 for plant metabolism) was mean-centered and weighed with the inverse of the standard deviation before performing this analysis to guarantee that all variables had the same weight when building the model. All statistical analyses were performed in IBM SPSS Statistics 26.

## 5. Conclusions

Although the effects of single N and W deficits have been well documented on morphological, physiological, and molecular mechanisms of tomato, the impact of combined N and water deficit is still far from being understood and explored. In a recent study, we showed that the combination of N and W clearly induces differential impacts on the redox homeostasis of young tomato plants, suggesting that significant metabolic alterations might be taking place. Here we moved one step forward, showing that tomato plants can employ N remobilization and osmoregulatory mechanisms as a coping strategy to combined N+W deficit. Additionally, the pattern of response seems to be quite distinct among different plant organs. Future work should address tomato responses to more severe levels of single and combined N+W stress, as well as explore more susceptible developmental stages.

## Figures and Tables

**Table 1:** Morphophysiological responses of tomato cv. Micro-Tom grown for 16 days under control (CTR; 10.5 mM N + 100% W), nitrogen deficit (N; 5.3 mM N + 100% W), water deficit (W; 10.5 mM N + 50% W) or combined nitrogen and water deficit (N+W; 5.3 mM N + 50% W). Abbreviations: DW = dry weight; Ψshoot = shoot water potential.

Parameters	CTR	N Deficit	W Deficit	N+W Deficit	*p*-Value
Total DW (g)	0.774 ± 0.051 ^a^	0.567 ± 0.040 ^b^	0.750 ± 0.028 ^a^	0.566 ± 0.037 ^b^	**0.0010**
Shoot DW (g)	0.592 ± 0.037 ^a^	0.429 ± 0.029 ^b^	0.536 ± 0.030 ^ab^	0.402 ± 0.028 ^b^	**0.0009**
Root DW (g)	0.182 ± 0.016 ^ab^	0.138 ± 0.012 ^b^	0.214 ± 0.013 ^a^	0.164 ± 0.011 ^ab^	**0.0035**
Shoot-to-Root Ratio (g/g dw)	3.65 ± 0.15 ^a^	3.14 ± 0.09 ^ab^	2.55 ± 0.22 ^bc^	2.46 ± 0.11 ^c^	**<0.0001**
Leaf Weight Ratio (g/g dw)	0.62 ± 0.01 ^a^	0.60 ± 0.01 ^a^	0.55 ± 0.02 ^b^	0.55 ± 0.01 ^b^	**0.0012**
Leaf Area (cm^2^)	61.29 ± 2.94 ^a^	42.08 ± 2.50 ^b^	58.94 ± 2.24 ^a^	39.40 ± 2.76 ^b^	**<0.0001**
Specific Leaf Area (cm^2^/g dw)	130 ± 9.91	125 ± 4.69	143 ± 5.42	125 ± 6.52	0.1092
Plant Height (cm)	9.00 ± 0.73	8.83 ± 0.48	10.67 ± 0.61	9.33 ± 0.56	0.1606
Internode Number	6.17 ± 0.40	6.00 ± 0.37	6.83 ± 0.40	5.83 ± 0.31	0.2728
Average Internode Length (cm)	1.48 ± 0.14	1.49 ± 0.10	1.56 ± 0.04	1.61 ± 0.11	0.7753
Root Length (cm)	23.5 ± 1.71	26.0 ± 2.12	26.4 ± 0.51	24.2 ± 2.85	0.7162
Chlorophyll Content (SPAD)	61.7 ± 0.42 ^a^	53.0 ± 1.28 ^b^	61.04 ± 0.55 ^a^	50.82 ± 0.98 ^b^	**0.0888**
Nitrogen Use Efficiency (g dw/g N applied)	14.9 ± 2.15 ^b^	26.4 ± 1.94 ^a^	17.0 ± 0.65 ^b^	25.7 ± 1.68 ^a^	**0.0001**
Nitrogen Accumulation (mg)	11.6 ± 1.03 ^b^	7.58 ± 0.79 ^c^	16.15 ± 0.88 ^a^	7.38 ± 0.44 ^c^	**0.0002**
Water Use Efficiency (mg dw/mL)	3.50 ± 0.12	3.73 ± 0.21	4.02 ± 0.13	3.46 ± 0.14	0.0682
Ψshoot (MPa)	−4.67 ± 0.40	−5.50 ± 0.18	−5.05 ± 0.59	−4.67 ± 0.42	0.4725

Data presented are mean ± standard error of the mean (SEM) (n = 3 for nitrogen use efficiency and nitrogen accumulation and n = 6 for all the other parameters). Different letters above indicate significant differences according to Tukey’s HSD test (*p* = 0.05).

**Table 2:** Primer sequences (Forward and Reverse) and annealing temperature (Tann) of target genes used for RT-qPCR analysis.

Gene (Accession Number)	Primer Sequence (5′-3′)		T_ann._
	Forward	Reverse	
*rcbL*	ATC TTG CTC GGG AAG GTA ATG	TCT TTC CAT ACC TCA CAA GCA G	55.9
*rcbS*	TGA GAC TGA GCA CGG ATT TG	TTT AGC CTC TTG AAC CTC AGC	55.7
*GS1* *NM_001319855.1*	ACA GCA CCA AGT CGA TGA GG	TGA TGT TGG CTG TTT CGT GC	58.8
*GS2.1* *NM_001323669.1*	TGC ATT GTC CAC TTA GTT GGT T	TTC AGC ACC ACA GAG CTC CA	57.7
*GS2.1* *NM_001323670.1*	TGC ATT GTC CAC TTA GGA GGT	CAC CAC AGA GCT CCA CAT CTT	56.2
*NR*	CAA GCA ATC CAT CTC CCA T	CAT CTC TGT ATC GTC TTC AGG A	53.3

## References

[1] Fernandes A., Machado J., Fernandes T., Vasconcelos M., Carvalho S. (2022). Water and nitrogen fertilization management in light of climate change: Impacts on food security and product quality. Plant Nutrition and Food Security in the Era of Climate Change.

[2] Esteban R., Ariz I., Cruz C., Moran J. (2016). Review: Mechanisms of ammonium toxicity and the quest for tolerance. Plant Sci..

[3] Liang L., Ridoutt B.G., Lal R., Wang D., Wu W., Peng P., Hang S., Wang L., Zhao G. (2019). Nitrogen footprint and nitrogen use efficiency of greenhouse tomato production in North China. J. Clean. Prod..

[4] Elbehri A. (2015). Climate Change and Food Systems: Global Assessments and Implications for Food Security and Trade.

[5] Coskun D., Britto D.T., Shi W., Kronzucker H.J. (2017). How plant root exudates shape the nitrogen cycle. Trends Plant Sci..

[6] Wang R., Min J., Kronzucker H.J., Li Y., Shi W. (2019). N and P runoff losses in China’s vegetable production systems: Loss characteristics, impact, and management practices. Sci. Total Environ..

[7] Coskun D., Britto D.T., Shi W., Kronzucker H.J. (2017). Nitrogen transformations in modern agriculture and the role of biological nitrification inhibition. Nat. Plants.

[8] Liu J., Ma K., Ciais P., Polasky S. (2016). Reducing human nitrogen use for food production. Sci. Rep..

[9] Schebesta H., Candel J.J.L. (2020). Game-changing potential of the EU’s Farm to Fork Strategy. Nat. Food.

[10] Heuvelink E., Okello R.C., Peet M., Giovannoni J.J., Dorais M. (2020). Tomato.

[11] De Cicco A. (2019). The Fruit and Vegetable Sector in the EU—A Statistical Overview Eurostat 2019.

[12] Sandhu R.K., Boyd N.S., Zotarelli L., Agehara S., Peres N. (2021). Effect of planting density on the yield and growth of intercropped tomatoes and peppers in Florida. HortScience.

[13] Du Y.-D., Niu W.-Q., Gu X.-B., Zhang Q., Cui B.-J. (2018). Water-and nitrogen-saving potentials in tomato production: A meta-analysis. Agric. Water Manag..

[14] Machado J., Fernandes A., Fernandes T., Heuvelink E., Vasconcelos M., Carvalho S. (2022). Drought and nitrogen stress effects and tolerance mechanisms in tomato: A review. Plant Nutrition and Food Security in the Era of Climate Change.

[15] Gonzalez-Dugo V., Durand J.-L., Gastal F. (2010). Water deficit and nitrogen nutrition of crops. A review. Agron. Sustain. Dev..

[16] Ding L., Lu Z., Gao L., Guo S., Shen Q. (2018). Is nitrogen a key determinant of water transport and photosynthesis in higher plants upon drought stress?. Front. Plant Sci..

[17] Szabados L., Savouré A. (2010). Proline: A multifunctional amino acid. Trends Plant Sci..

[18] Hayat S., Hayat Q., Alyemeni M.N., Wani A.S., Pichtel J., Ahmad A. (2012). Role of proline under changing environments: A review. Plant Signal. Behav..

[19] Bradford M.M. (1976). A rapid and sensitive method for the quantitation of microgram quantities of protein utilizing the principle of protein-dye binding. Anal. Biochem..

[20] Kavoosi G., Balotf S., Eshghi H., Hasani H. (2014). Analysis of nitrate reductase mRNA expression and nitrate reductase activity in response to nitrogen supply. Mol. Biol. Res. Commun..

[21] Lemaître T., Gaufichon L., Boutet-Mercey S., Christ A., Masclaux-Daubresse C. (2008). Enzymatic and metabolic diagnostic of nitrogen deficiency in Arabidopsis thaliana Wassileskija accession. Plant Cell Physiol..

[22] Nacry P., Bouguyon E., Gojon A. (2013). Nitrogen acquisition by roots: Physiological and developmental mechanisms ensuring plant adaptation to a fluctuating resource. Plant Soil.

[23] Plett D.C., Ranathunge K., Melino V.J., Kuya N., Uga Y., Kronzucker H.J. (2020). The intersection of nitrogen nutrition and water use in plants: New paths toward improved crop productivity. J. Exp. Bot..

[24] Cao X., Zhong C., Zhu C., Zhu L., Zhang J., Wu L., Jin Q. (2018). Ammonium uptake and metabolism alleviate PEG-induced water stress in rice seedlings. Plant Physiol. Biochem..

[25] Végh K.R. (1991). Effect of soil water and nutrient supply on root characteristics and nutrient uptake of plants. Developments in Agricultural and Managed Forest Ecology.

[26] Sánchez-Rodríguez E., Rubio-Wilhelmi M., Blasco B., Constán-Aguilar C., Romero L., Ruiz J. (2011). Variation in the use efficiency of N under moderate water deficit in tomato plants (*Solanum lycopersicum*) differing in their tolerance to drought. Acta Physiol. Plant..

[27] Sánchez-Rodríguez E., del Mar Rubio-Wilhelmi M., Ríos J.J., Blasco B., Rosales M.Á., Melgarejo R., Romero L., Ruiz J.M. (2011). Ammonia production and assimilation: Its importance as a tolerance mechanism during moderate water deficit in tomato plants. J. Plant Physiol..

[28] Zhou R., Yu X., Ottosen C.-O., Rosenqvist E., Zhao L., Wang Y., Yu W., Zhao T., Wu Z. (2017). Drought stress had a predominant effect over heat stress on three tomato cultivars subjected to combined stress. BMC Plant Biol..

[29] Hussain H.A., Hussain S., Khaliq A., Ashraf U., Anjum S.A., Men S., Wang L. (2018). Chilling and drought stresses in crop plants: Implications, cross talk, and potential management opportunities. Front. Plant Sci..

[30] Sousa B., Rodrigues F., Soares C., Martins M., Azenha M., Lino-Neto T., Santos C., Cunha A., Fidalgo F. (2022). Impact of Combined Heat and Salt Stresses on Tomato Plants—Insights into Nutrient Uptake and Redox Homeostasis. Antioxidants.

[31] Machado J., Vasconcelos M.W., Soares C., Fidalgo F., Heuvelink E., Carvalho S.M.P. (2023). Enzymatic and Non-Enzymatic Antioxidant Responses of Young Tomato Plants (cv. Micro-Tom) to Single and Combined Mild Nitrogen and Water Deficit: Not the Sum of the Parts. Antioxidants.

[32] Ruggiero A., Punzo P., Van Oosten M.J., Cirillo V., Esposito S., Costa A., Maggio A., Grillo S., Batelli G. (2022). Transcriptomic and splicing changes underlying tomato responses to combined water and nutrient stress. Front. Plant Sci..

[33] Shao H.-B., Chu L.-Y., Jaleel C.A., Zhao C.-X. (2008). Water-deficit stress-induced anatomical changes in higher plants. Comptes Rendus Biol..

[34] Nemeskéri E., Neményi A., Bőcs A., Pék Z., Helyes L. (2019). Physiological Factors and their Relationship with the Productivity of Processing Tomato under Different Water Supplies. Water.

[35] Khan M., Khan M.J., Ahmad S., Ali A., Khan N., Fahad M.A. (2020). Effect of different nitrogen doses and deficit irrigation on nitrogen use efficiency and growth parameters of tomato crop under drip irrigation system. Sarhad J. Agric..

[36] Sibomana I., Aguyoh J., Opiyo A. (2013). Water stress affects growth and yield of container grown tomato (*Lycopersicon esculentum* Mill) plants. GJBB.

[37] Khan S.H., Arsalan K., Uzma L., Shah A.S., Khan M.A., Muhammad B., Ali M.U. (2015). Effect of drought stress on tomato cv. Bombino. J. Food Process. Technol..

[38] Moles T.M., Mariotti L., De Pedro L.F., Guglielminetti L., Picciarelli P., Scartazza A. (2018). Drought induced changes of leaf-to-root relationships in two tomato genotypes. Plant Physiol. Biochem..

[39] Selim A.-F.H., El-Nady M.F. (2011). Physio-anatomical responses of drought stressed tomato plants to magnetic field. Acta Astronaut..

[40] Pokluda R., Petříková K., Abdelaziz M.E., Jezdinský A. (2010). Effect of water stress on selected physiological characteristics of tomatoes. Acta Univ. Agric. Silvic. Mendel. Brun..

[41] Scholberg J., McNeal B.L., Boote K.J., Jones J.W., Locascio S.J., Olson S.M. (2000). Nitrogen stress effects on growth and nitrogen accumulation by field-grown tomato. Agron. J..

[42] Vos J., Van Der Putten P., Birch C. (2005). Effect of nitrogen supply on leaf appearance, leaf growth, leaf nitrogen economy and photosynthetic capacity in maize (*Zea mays* L.). Field Crops Res..

[43] Cannella D., Möllers K., Frigaard N.-U., Jensen P., Bjerrum M., Johansen K., Felby C. (2016). Light-driven oxidation of polysaccharides by photosynthetic pigments and a metalloenzyme. Nat. Commun..

[44] Baquedano F., Castillo F. (2006). Comparative ecophysiological effects of drought on seedlings of the Mediterranean water-saver Pinus halepensis and water-spenders Quercus coccifera and Quercus ilex. Trees.

[45] Bassi D., Menossi M., Mattiello L. (2018). Nitrogen supply influences photosynthesis establishment along the sugarcane leaf. Sci. Rep..

[46] Ashraf M., Harris P.J. (2013). Photosynthesis under stressful environments: An overview. Photosynthetica.

[47] Tamburino R., Vitale M., Ruggiero A., Sassi M., Sannino L., Arena S., Costa A., Batelli G., Zambrano N., Scaloni A. (2017). Chloroplast proteome response to drought stress and recovery in tomato (*Solanum lycopersicum* L.). BMC Plant Biol..

[48] Soval-Villa M., Wood C., Guertal E. (2002). Tomato leaf chlorophyll meter readings as affected by variety, nitrogen form, and nighttime nutrient solution strength. J. Plant Nutr..

[49] Safavi-Rizi V., Franzaring J., Fangmeier A., Kunze R. (2018). Divergent N deficiency-dependent senescence and transcriptome response in developmentally old and young *Brassica napus* leaves. Front. Plant Sci..

[50] Zakari S.A., Zaidi S.H.R., Sunusi M., Dauda K.D. (2021). Nitrogen deficiency regulates premature senescence by modulating flag leaf function, ROS homeostasis, and intercellular sugar concentration in rice during grain filling. J. Genet. Eng. Biotechnol..

[51] Sánchez-Rodríguez E., Moreno D.A., Ferreres F., del Mar Rubio-Wilhelmi M., Ruiz J.M. (2011). Differential responses of five cherry tomato varieties to water stress: Changes on phenolic metabolites and related enzymes. Phytochemistry.

[52] Khapte P., Kumar P., Burman U., Kumar P. (2019). Deficit irrigation in tomato: Agronomical and physio-biochemical implications. Sci. Hortic..

[53] Taiz L., Zeiger E., Møller I.M., Murphy A. (2015). Plant Physiology and Development.

[54] Pirasteh-Anosheh H., Saed-Moucheshi A., Pakniyat H., Pessarakli M. (2016). Stomatal responses to drought stress. Water Stress Crop Plants Sustain. Approach.

[55] Blum A. (2017). Osmotic adjustment is a prime drought stress adaptive engine in support of plant production. Plant Cell Environ..

[56] Nahar K., Ullah S. (2017). Fruit Quality and Osmotic Adjustment of Four Tomato Cultivars under Drought Stress. Asian J. Soil Sci. Plant Nutr..

[57] Kosová K., Vítámvás P., Urban M.O., Prášil I.T., Renaut J. (2018). Plant abiotic stress proteomics: The major factors determining alterations in cellular proteome. Front. Plant Sci..

[58] Qazi H.A., Jan N., Ramazan S., John R. (2019). Protein modification in plants in response to abiotic stress. Protein Modificomics.

[59] Alscher R.G., Erturk N., Heath L.S. (2002). Role of superoxide dismutases (SODs) in controlling oxidative stress in plants. J. Exp. Bot..

[60] Kapilan R., Vaziri M., Zwiazek J.J. (2018). Regulation of aquaporins in plants under stress. Biol. Res..

[61] Afzal Z., Howton T., Sun Y., Mukhtar M.S. (2016). The roles of aquaporins in plant stress responses. J. Dev. Biol..

[62] Yang X., Lu M., Wang Y., Wang Y., Liu Z., Chen S. (2021). Response Mechanism of Plants to Drought Stress. Horticulturae.

[63] Urbanczyk-Wochniak E., Fernie A.R. (2005). Metabolic profiling reveals altered nitrogen nutrient regimes have diverse effects on the metabolism of hydroponically-grown tomato (*Solanum lycopersicum*) plants. J. Exp. Bot..

[64] Khavari-Nejad R.A., Najafi F., Tofighi C. (2009). Diverse responses of tomato to N and P deficiency. Int. J. Agric. Biol..

[65] Coruzzi G.M., Zhou L. (2001). Carbon and nitrogen sensing and signaling in plants: Emerging ‘matrix effects’. Curr. Opin. Plant Biol..

[66] Martin T., Oswald O., Graham I.A. (2002). Arabidopsis seedling growth, storage lipid mobilization, and photosynthetic gene expression are regulated by carbon: Nitrogen availability. Plant Physiol..

[67] Aoyama S., Huarancca Reyes T., Guglielminetti L., Lu Y., Morita Y., Sato T., Yamaguchi J. (2014). Ubiquitin ligase ATL31 functions in leaf senescence in response to the balance between atmospheric CO_2_ and nitrogen availability in Arabidopsis. Plant Cell Physiol..

[68] Foyer C.H., Valadier M.-H., Migge A., Becker T.W. (1998). Drought-induced effects on nitrate reductase activity and mRNA and on the coordination of nitrogen and carbon metabolism in maize leaves. Plant Physiol..

[69] Chen D., Wang S., Xiong B., Cao B., Deng X. (2015). Carbon/nitrogen imbalance associated with drought-induced leaf senescence in Sorghum bicolor. PLoS ONE.

[70] Ferrario-Méry S., Valadier M.-H., Foyer C.H. (1998). Overexpression of nitrate reductase in tobacco delays drought-induced decreases in nitrate reductase activity and mRNA. Plant Physiol..

[71] Robredo A., Pérez-López U., Miranda-Apodaca J., Lacuesta M., Mena-Petite A., Muñoz-Rueda A. (2011). Elevated CO_2_ reduces the drought effect on nitrogen metabolism in barley plants during drought and subsequent recovery. Environ. Exp. Bot..

[72] Fukutoku Y., Yamada Y. (1984). Sources of proline-nitrogen in water-stressed soybean (*Glycine max*). II. Fate of 15N-labelled protein. Physiol. Plant..

[73] Kaiser W.M., Huber S.C. (2001). Post-translational regulation of nitrate reductase: Mechanism, physiological relevance and environmental triggers. J. Exp. Bot..

[74] Lacrampe N., Lopez-Lauri F., Lugan R., Colombié S., Olivares J., Nicot P.C., Lecompte F. (2020). Regulation of sugar metabolism genes in the nitrogen-dependent susceptibility of tomato stems to Botrytis cinerea. Ann. Bot..

[75] Galangau F., Daniel-Vedele F., Moureaux T., Dorbe M.-F., Leydecker M.-T., Caboche M. (1988). Expression of leaf nitrate reductase genes from tomato and tobacco in relation to light-dark regimes and nitrate supply. Plant Physiol..

[76] Masclaux-Daubresse C., Reisdorf-Cren M., Orsel M. (2008). Leaf nitrogen remobilisation for plant development and grain filling. Plant Biol..

[77] Diaz C., Lemaître T., Christ A., Azzopardi M., Kato Y., Sato F., Morot-Gaudry J.-F., Le Dily F., Masclaux-Daubresse C. (2008). Nitrogen recycling and remobilization are differentially controlled by leaf senescence and development stage in Arabidopsis under low nitrogen nutrition. Plant Physiol..

[78] Malagoli P., Laine P., Rossato L., Ourry A. (2005). Dynamics of nitrogen uptake and mobilization in field-grown winter oilseed rape (*Brassica napus*) from stem extension to harvest. II. An 15N-labelling-based simulation model of N partitioning between vegetative and reproductive tissues. Ann. Bot..

[79] Thompson A.R., Doelling J.H., Suttangkakul A., Vierstra R.D. (2005). Autophagic nutrient recycling in Arabidopsis directed by the ATG8 and ATG12 conjugation pathways. Plant Physiol..

[80] Wingler A., Roitsch T. (2008). Metabolic regulation of leaf senescence: Interactions of sugar signalling with biotic and abiotic stress responses. Plant Biol..

[81] Hunt R. (2012). Basic Growth Analysis: Plant Growth Analysis for Beginners.

[82] Carvalho D.R.A., Vasconcelos M.W., Lee S., Vreugdenhil D., Heuvelink E., Carvalho S.M.P. (2017). Moderate salinity improves stomatal functioning in rose plants grown at high relative air humidity. Environ. Exp. Bot..

[83] Bates L.S., Waldren R.P., Teare I. (1973). Rapid determination of free proline for water-stress studies. Plant Soil.

[84] Fidalgo F., Freitas R., Ferreira R., Pessoa A.M., Teixeira J. (2011). Solanum nigrum L. antioxidant defence system isozymes are regulated transcriptionally and posttranslationally in Cd-induced stress. Environ. Exp. Bot..

[85] Mills H., Jones J.B. (1996). Plant Analysis Handbook II.

[86] Kaiser W.M., Brendle-Behnisch E. (1991). Rapid modulation of spinach leaf nitrate reductase activity by photosynthesis: I. Modulation in vivo by CO_2_ availability. Plant Physiol..

[87] Shapiro B.M., Stadtman E. (1970). [130] Glutamine synthetase (*Escherichia coli*). Methods in Enzymology.

[88] Mariz-Ponte N., Mendes R.J., Sario S., Correia C.V., Correia C.M., Moutinho-Pereira J., Melo P., Dias M.C., Santos C. (2021). Physiological, biochemical and molecular assessment of UV-A and UV-B supplementation in *Solanum lycopersicum*. Plants.

[89] Jin C.W., Du S.T., Zhang Y.S., Lin X.Y., Tang C.X. (2009). Differential regulatory role of nitric oxide in mediating nitrate reductase activity in roots of tomato (*Solanum lycocarpum*). Ann. Bot..

[90] Livak K.J., Schmittgen T.D. (2001). Analysis of relative gene expression data using real-time quantitative PCR and the 2^−ΔΔCT^ method. Methods.

[91] Vaerman J., Saussoy P., Ingargiola I. (2004). Evaluation of real-time PCR data. J. Biol. Regul. Homeost. Agents.

